# Influence of scat ageing on the gut microbiome: how old is too old?

**DOI:** 10.1186/s12864-023-09520-0

**Published:** 2023-07-31

**Authors:** Alejandro Oliveros, Julien Terraube, Alexis L. Levengood, Daniel Powell, Céline H. Frère

**Affiliations:** 1 The School of Biological Sciences, The University of Queensland, St Lucia, QLD Australia; 2 Vulture Conservation Foundation, Wuhrstrasse 12, Zürich, CH-8003 Switzerland; 3 School of Science, Technology and Engineering, University of the Sunshine Coast, Sippy Downs, QLD Australia

**Keywords:** Conservation, Metagenomics, Faecal microbiome, Fungi, Koala, Non-invasive sampling, Time Series

## Abstract

**Background:**

The study of the host-microbiome by the collection of non-invasive samples has the potential to become a powerful tool for conservation monitoring and surveillance of wildlife. However, multiple factors can bias the quality of data recovered from scats, particularly when field-collected samples are used given that the time of defecation is unknown. Previous studies using scats have shown that the impact of aerobic exposure on the microbial composition is species-specific, leading to different rates of change in microbial communities. However, the impact that this aging process has on the relationship between the bacterial and fungal composition has yet to be explored. In this study, we measured the effects of time post-defecation on bacterial and fungal compositions in a controlled experiment using scat samples from the endangered koala (*Phascolarctos cinereus*).

**Results:**

We found that the bacterial composition remained stable through the scat aging process, while the fungal composition did not. The absence of an increase in facultative anaerobes and the stable population of obligate anaerobic bacteria were likely due to our sampling from the inner portion of the scat. We report a cluster of fungal taxa that colonises scats after defecation which can dilute the genetic material from the autochthonous mycoflora and inhibit recovery.

**Conclusion:**

We emphasize the need to preserve the integrity of scat samples collected in the wild and combat the effects of time and provide strategies for doing so.

**Supplementary Information:**

The online version contains supplementary material available at 10.1186/s12864-023-09520-0.

## Introduction

Research has shown that gut microbiota and their metabolites influence the overall health of animals, including physiology, nutrition, immunology, and behaviour (e.g., [1]). It is understood that the composition of the microbiome (bacteria, archaea, viruses, fungi, and protists) is shaped by the genes and the environment of animals [2, 3]. Together these factors contribute to maintaining homeostasis, which is paramount to host fitness [4, 5]. An imbalance in the composition of the bacterial gut communities (i.e., dysbiosis) can be linked to numerous host diseases (e.g., cancer, malnutrition, and increased severity of SARS-CoV-2 infection) [6]. Changes in fungal abundance can also compromise animals’ health [7, 8]. For instance, overgrowth of *Candida albicans* is a major cause of morbidity and death in critical care settings not only for humans [9] but also for other mammals such as koalas (Australia Zoo Wildlife Hospital, personal communication). As such, the study of gut microbiomes is becoming an essential component of conservation biology [2, 4].

Health parameters are increasingly being used to monitor populations, yet they should accurately describe the characteristics of the specific population (health status, fitness, or population trends). Understanding the drivers of host-microbiome variations can inform wildlife conservation and management by identifying baseline population characteristics. Studies such as Littleford-Colquhoun et al.[10] highlight the need to use microbiome composition and structure alongside traditional indices in future research, potentially identifying high-quality habitats for threatened species.

Building on the importance of microbiome research in conservation and the role of health parameters, diet plays a vital role in shaping the gut microbiome [11–16], making microbiome studies informative for translocating or reintroducing endangered species[10, 16–18]. As gut bacterial communities have coevolved with their host to maximize digestion [16–18], translocated individuals may need to adjust their microbiome to a new diet post-translocation. Feeding subjects with a diet from the new habitat while in care can be highly beneficial [16]. Yao et al. [16] demonstrated that monitoring host’s microbiota before, during, and after translocation can guide adaptation to a new diet, improving translocation or reintroduction projects and individuals’ health/adaptation capacity in the wild.

As studies into host-microbe communities uncover factors that drive the population’s health and fitness, incorporating microbial analysis into conservation monitoring and surveillance becomes increasingly valuable. Previously, accessing a host’s microbiome relied purely upon invasive methods [19, 20], which can be more challenging than acquiring other types of baseline data on endangered individuals. As a result, non-invasive sampling, where DNA is obtained from the species’ scats, feathers, or hair [19, 20], has risen in popularity over the last decade. This sampling method is facilitating a more cost-effective, accessible, large-scale approach for the monitoring and surveillance of the current state of wildlife populations [15, 20–23]. This combination of non-invasive sampling and molecular analysis of the host microbiome in the wild, termed conservation metagenomics [22], offers promising ways of monitoring wild populations using non-invasive sampling methods, generating an accessible baseline even for threatened species, and uncovering quantifiable measurements such as population health, density, and dispersal [15, 22, 23]. Thus, metagenomics provides highly informative data to assess the state of a population and the effects of conservation strategies being implemented.

Despite the advantages of using non-invasive samples like faecal matter, this approach presents specific challenges that must be addressed. This is because multiple factors can bias the quality of the non-invasive metagenomic sample. There is an ongoing effort to establish proper methods and best practices to ensure the quality of the sample, such as sample collection methods, freezing methods, sequencing methods, and DNA extraction [24, 25]. Yet, it seems that a crucial step has been overlooked; if we aim to develop this approach, it is essential to acknowledge that DNA recovered from non-invasive samples are often poor quality due to environmental degradation [21]. The integrity of a scat sample will degrade with time due to the environmental forces changing the characteristics of the sample. This presents an important challenge for non-invasive metagenomic sampling as a tool for conservation since it is impossible to know for how long a sample has been exposed to the environment before collection.

Although little is known of the effects of the exposed sample to environmental degradation, few studies have started investigating the impact of scat sample age in the study of the gut microbiome in wildlife [26–29]. These studies argue that bacterial communities in the scat sample will likely change from anaerobic to aerobic as the sample is exposed to air [26–29]. For instance, Wong et al. [27] showed that the bacterial composition in faecal cowpat samples shifted after only two days as *Clostridia* and *Bacteroidia* disappeared from the faecal cowpats, given that they are obligate anaerobes. The authors also documented an increase in Actinobacteria and Proteobacteria over time as they are facultative anaerobes. Together, these studies argue that the susceptibility of scat samples to the impact of oxygen exposure will be host species-specific [26, 27, 29], given that sample exposure to oxygen will vary depending on its size and shape. Thus, the compositional changes in the microbiome of the scat samples need to be evaluated for each host species before accurate interpretations of a scat sample can be used for monitoring purposes [26, 27, 29]. For this reason, there is a need to ensure that differences between individuals or populations are not a consequence of the age of the scat sample post defecation. All studies thus far on wildlife have only measured the effect of scat sample age on bacterial communities. If we take into account that the fungal sub-kingdom is known as one of the most abundant and diverse decomposers on earth [8], it can be considered fundamental to include the mycobiome in our analyses of how scat samples’ age influences the composition. Furthermore, expanding the microbial toolkit to other kingdoms is essential as these taxa may also play a vital role in host fitness [4, 7].

Koalas have historically been costly and challenging to study due to their cryptic behaviour and low density. However, while they may be hard to find, they are known to defecate frequently. This makes studying the koala gut microbiome through the acquisition of non-invasive faecal samples a potentially cost-effective approach. Incorporating the study of the gut microbiome to koala conservation could assist its management in several ways. First, with loss of habitat and its fragmentation, conservation programs are increasingly looking at translocation as an important means to conserve koalas. Given that each koala has a gut microbiome highly specialised to its local diet [13], its study would be very helpful in ensuring translocations are successful. Second the microbiome has been shown to contain important information about population substructures which could not be identified via population genetic analyses of the koala DNA [10]. Third, the faecal microbiome of koalas may contain important health bio-markers which would help in (a) identifying populations (or individuals) in need of medical intervention and (b) build a better understanding of the link between environmental conditions and health allowing us to identify high-quality habitats for koalas.

To address these challenges, our study aims to measure the effects of scat aging on the koala microbiome, as the quality of metagenomic samples depends on the stability of the microbial composition and abundance. Understanding these changes is critical, as any alterations introduced by the scat aging process could impede the use of scat samples as reliable proxies for the koala gut microbiome. This study aims to track changes in the bacterial and fungal composition of koala scats over time, providing insights into the factors affecting microbial communities in scat samples. We predicted that the microbial communities contained in the koala scats would change in composition over time and that the change would be more pronounced as the scat aged.

## Materials and methods

### Experimental design and sample processing

Fresh koala scats were collected from the cages of five individual koalas held for veterinary checks at Endeavour Veterinary Ecology Clinic in Toorbul, Queensland under animal ethics approval from the University of the Sunshine Coast. The koalas came from different locations, which we note may influence the microbial composition of their scat microbiomes. Additionally, it is important to note that Koala 1 had been exposed to antibiotics (chloramphenicol) for Chlamydia treatment, which may also have affected its microbiome. Further details on the koalas’ locations and health notes can be found in Table 1.

**Table 1:** Metadata table: In November, 2020, scats were collected in 50ml centrifuge tubes and covered with gauze to allow the escape of volatile compounds

Koala ID	Location	Cage time	Sex	Health Notes
1	Hidden Vale, QLD	12 pm	F	Treated for Chlamydia with chloramphenicol. Last dose on 19/11/2020
2	Petrie, QLD	3 pm	M	Held for a vet exam. No signs of disease
3	Gympie, QLD	5 pm	F	Female mother with joey. Held for a vet exam
4	Petrie, QLD	5 pm	M	Has a broken arm and is on pain relief. No sign of disease
5	Gympie, QLD	3 pm	M	No signs of disease. Held for a vet exam

**Fig. 1:**
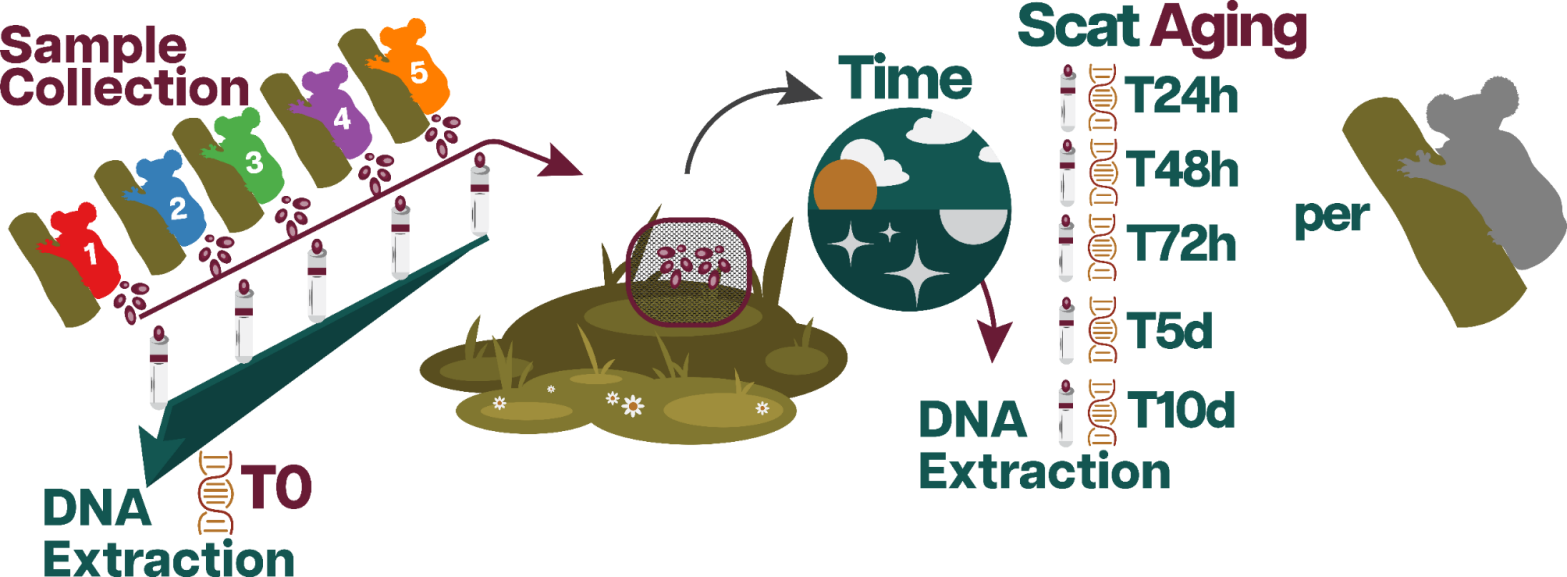
Diagram of scat aging experiment: Fresh koala scat samples were collected from five koalas. One scat pellet for each koala was processed for DNA extraction immediately after defecation. Five scat pellets for each koala were then placed on toothpicks in outdoor meshed enclosures aged under natural conditions. After the following time points, one scat pellet for each koala was collected and processed for DNA extractions at: 24 h, 48 h, 72 h,5 days, and 10 days

Following transport to campus in sterile tubes open to the air, one scat pellet from each koala was processed for DNA extraction immediately. An additional five scat pellets for each koala were then mounted on toothpicks, suspended on Styrofoam trays, and placed into meshed enclosures (to prevent insects or other animal activity from damaging the scat samples) and aged under natural conditions in a remnant patch of forest on campus (Supplementary Fig. 1). One scat pellet from each koala was recovered and processed at 24 h, 48 h, 72 h, 5 days, and 10 days post initial collection (Fig. 1). This resulted in six time-points for five koalas, totalling 30 scat samples. The core of the scat sample was then extracted to minimize the impact of oxygen exposure using the QIAamp PowerFecal Pro DNA Kit (Qiagen), following the manufacturer’s protocol, with the following variations: after adding CD1 buffer, samples were incubated at 65° C for 1 h and vortexed for 7 min at maximum speed using Genie 2 Vortex Mixer (Scientific Industries). The DNA was eluted in a final volume of 100 µl of C6 buffer and stored at -80 °C.

### Sequencing

DNA samples were sequenced targeting both the bacterial and fungal communities. To identify bacteria, the V3 to V4 region of the 16 S rRNA gene was targeted using the universal primers 341 F (CCTAYGGGRBGCASCAG) and 806R (GGACTACNNGGGTATCTAAT) [30]. To identify fungal communities, the internal transcribed spacer (ITS)1-F and ITS2 regions were targeted using the forward primer (CTTGGTCATTTAGAGGAAGTAA) and the reverse primer (GCTGCGTTCTTCATCGATGC) [31]. Library preparation was performed using Nextera XT dual-index barcodes using a two-step protocol to firstly amplify the 16 S/ITS region then add the indexes. Libraries were then pooled in equimolar ratios. Bacterial and fungal libraries were sequenced on separate flow cells on the Illumina MiSeq platform using 300 bp paired-end reads. All library preparation steps and sequencing was undertaken at the Australian Genome Research Facility (AGRF). Basecalling was performed in real time by the MiSeq Control Software (v3.1.0.13) and Real Time Analysis (v1.18.54.4), running on the instrument computer. Then the Illumina DRAGEN BCL Convert 07.021.624.3.10.8 pipeline was used to generate the sequence data. The demultiplexed raw reads were primer trimmed and quality filtered using the Cutadapt plugin followed by denoising with DADA2 [32] (via q2-dada2). Quality control metrics are shown in Supplementary Tables 1 and 2.

### Data analysis

#### Diversity analysis

Diversity analysis was performed using QIIME (v2021.4.0) [33]. For fungi (ITS1 and ITS2), we rarefied to 5000 reads per sample. For bacteria (V3 and V4), we rarefied to 76,000 reads per sample. This was done considering rarefaction curves using the diversity alpha-rarefaction plugin [33]. Our goal was to minimise data loss while maintaining a sufficient number of reads. Once the data was filtered, we measured alpha and beta diversity metrics.

Alpha diversity was estimated to assess the diversity of microbial communities within each scat pellet sample. The alpha diversity measurements for both bacteria and fungi were evaluated based on observed Amplicon Sequence Variants (ASVs), representing ASV richness, Pielou’s Evenness, and Shannon indexes using the QIIME diversity core-metrics plugin [33, 34]. To visualize how scat aging influenced microbial diversity over time, Shannon Entropy plots were generated using the QIIME longitudinal volatility plugin [33]. We performed the Friedman test, a nonparametric statistical test for repeated measurements, to assess the impact of time on the Shannon index.

To identify differences in microbial composition among scat samples, beta diversity was calculated. This was achieved by analyzing patterns in principal-coordinate analysis (PCoA) plots based on Jaccard and Bray-Curtis distances. The diversity core-metrics plugin of QIIME, as described by Bolyen et al. [33], was used to generate these plots using non-phylogenetic metrics. The reason for this is that the ITS region used to sample the fungal community is not appropriate for phylogenetic analysis [35]. However, we also generated phylogenetic metrics (unweighted and weighted UniFrac) for the bacterial community. These results can be found in the Supplementary Fig. 2. To generate the phylogenetic metrics, we constructed a phylogenetic tree using QIIME fragment-insertion plugin [33] based on Silva 128 SEPP reference database download from https://docs.qiime2.org/2022.2/data-resources/ [32, 36].

#### Effect of time on bacterial beta diversity metrics

To better understand and describe these dynamics, it is important to define our sample and establish clear terminology for comparing differences within and between koalas. A sample is defined as a scat pellet microbial community extraction. Each koala provided six scat pellets, with one processed at T0 and the others allowed to age and processed at different times. Moving forward, we will use the term “inter-koala differences” to denote the differences between all time points related to one koala in comparison to other koalas. When discussing a specific time, we will specify it such as “inter-koala differences at T0.“ On the other hand, “intra-koala differences” will be used to describe the changes observed in a particular koala’s scat pellet microbial communities across different time points.

As part of our study, we analyzed the impact of time on the first five axes of Principal Coordinates Analysis (PCoA) in all four beta diversity metrics measured in bacteria. These first five axes of PCoA are independent of each other and are arranged based on the variance they represent, with the first axis capturing the greatest variance. To determine the effect of time on these axes, we first calculated all possible absolute inter-koala differences at T0 which represented the “true differences”. We then computed the absolute difference between each koala at T0 and the remaining time points, which represented intra-koala differences with T0. Only when the intra-koala differences were greater than the inter-koala differences at T0, we consider time to have an impact on an axis. By comparing the true inter-koala differences with the variance introduced by time, we were able to evaluate the effect of time on these axes in all four beta diversity metrics.

We also performed the Friedman test on the same five axes of PCoA in all four beta diversity metrics to test for the significance of time in each axis, followed by a post hoc analysis comparing ranks between T0 and the remaining time points. These methods aim to discern the differential impacts of time on various aspects of the microbial communities. Each metric reveals different aspects of the bacterial communities present in the samples [37]. By evaluating the effect of time on these distinct beta diversity metrics - presence/absence metrics like Jaccard, abundance metrics like Bray-Curtis, and phylogenetic metrics like UniFrac - we can be better equipped to detect nuanced shifts in the microbial community structure during the scat aging process. Presence/absence metrics are sensitive to changes in which ASVs are present, while abundance metrics provide insights into variations in the relative quantities of these members. Phylogenetic metrics add another layer of complexity by considering the evolutionary relationships between these members. The differential responses of these metrics to aging give us a holistic view of the dynamics at play within the microbial communities of koala scats.

ASV relative abundance analysis was undertaken to track changes through time. To do so, we normalised abundance data to reads per million using the raw reads (input reads, Supplementary Table 1), which prevents the loss of data that occurs through rarefying. To assess the variation in ASVs (presence/absence and abundance) for both intra-koala differences and inter-koala differences, we utilized R (v4.1.0) [38]. In addition to the basic statistical tools that R provides, we also used the package *microbiome* [39]. Our objective was to evaluate the stability of ASVs over time, as well as the variation in their abundance for both intra-koala differences and inter-koala differences. These results were compared with the alpha and beta diversity profiles to check for consistency between the two analyses. We calculated the logarithmic base 10 ratio of anaerobes to facultative anaerobes of the 27 (95% of the total reads) most abundant bacteria at the family taxonomic level. This was done by assigning a respiration profile to each taxonomic family (Supplementary Table 3) with the exception of *Oxalobacteraceae*, which present an ambiguous respiratory profile ranging from obligate aerobes to obligate anaerobes.

### Taxonomic classification

#### Retrieval and utilization of bacterial databases

Several approaches were employed to assign taxonomy to each ASV sequence. For bacteria, the 16 S V3 and V4 ASVs were classified against three databases: [1] SILVA; [2] GTDB; [3] NCBI nucleotide database. QIIME formatted SILVA reference sequence (silva-138-99-seqs.qza) and taxonomy (silva-138-99-tax.qza) were downloaded from https://docs.qiime2.org/2021.8/data-resources/ [40]. We also used the GTDB FASTA file containing 16 S rRNA sequences (ssu_all_r202.tar.gz) from https://data.gtdb.ecogenomic.org/releases/release202/202.0/genomic_files_all/.

#### Retrieval and utilization of fungal databases

For fungi, the ITS ASVs were classified against three databases: [1] UNITE; [2] RefSeq; and [3] NCBI nucleotide database. The QIIME release UNITE database was downloaded from 10.15156/BIO/1264708. For this analysis, we used version 8 released on 10/05/2021 clustered at 99% identity. After downloading the FASTA file and the taxonomy rank attached to the accession numbers file, we used a customized R script to remove repeated sequences and replace unidentified fungi in the UNITE database with accession numbers. These were imported to QIIME. The RefSeq target loci fungal ITS database was downloaded from https://ftp.ncbi.nlm.nih.gov/refseq/TargetedLoci/Fungi/. We used a customized R script to remove repetitive sequences and transform these files to QIIME-compatible format for taxonomic classification. This script can be found in the Supplementary Data.

#### Identifying bacteria and fungi through NCBI nucleotide database by a blast search

For BLAST searches against the NCBI nucleotide database, ASVs were classified to the first hit of the BLAST search and further filtered if that hit did not correspond to the ITS region for fungi and 16 S V3 and V4 for bacteria. The percentage identity was kept as a record of trust in taxonomic classification. The BLAST search was performed for both bacteria and fungi. We also used several QIIME plugins to perform the taxonomic classification: (1) classify-consensus-blast [34]; (2) classify-consensus-vsearch [41]; (3) the plugin feature-classifier used to train a Naive-Bayes classifier against a specific database with subsequent amplified ASV classification [42]; and (4) classify-hybrid-vsearch-sklearn [33] using default and customized settings to classify the fungal ASVs, while only using the former for bacteria ASVs. The settings chosen for the different plugins can be found in the Supplementary Data. All plugins were used in accordance with QIMME documentation website https://docs.qiime2.org/2021.8/.

#### Taxonomic classification of bacteria and fungi: understanding trust scores

The above taxonomic classification tools were applied against each database except for the NCBI database. A customized R script was then used to define a definitive taxonomic rank for each ASV (from subkingdom to species, if possible), plus a trust score, which was done against each database to check how many classification tools were in agreement. This trust score was computed by assigning a weight to each taxonomic tool and then multiplying it by the confidence score given by the QIIME plugins. All the tools received a weight of 1 except for the Naive-Bayes classifier, which received 0.5 based on the poor performance experienced using this tool. For fungi, the five classification tools were used to normalize the final trust score into a 0 to 1 scale, and the sum of scores was divided by the possible max score of 4.5. For bacteria, we only used four tools; therefore, the final trust score was normalized dividing by 3.5. The final trust score represents the level of confidence that the chosen final classification had. However, if the classification tool failed to assign a taxonomic rank, the trust score was set to 0, and the taxonomic rank was categorized as “Unassigned”. The final taxa ranks, and their final trust score were assigned taking into account the highest frequency taxonomic ranks among tools. If only one tool could categorize a taxonomic rank for an ASV, then the same taxonomic rank and its score were considered the final taxonomic classification. In case of a tie between two conflicting taxonomic ranks with the same frequencies among tools, the one with the highest aggregating score was chosen. However, if the aggregating score between these two were the same, then the classify-consensus-blast result was assigned, and The final trust score was determined by adding up the number of tools that matched with this particular tool.

#### Clustering of the fungal ASVs to enhance classification

Even though we classified fungi against three databases and used five different tools to do so, the certainty and amount of taxonomically classified ASVs were low. To improve this, we performed clustering analysis using VSEARCH [41]. The representative fungi ASVs were clustered by identity coverage at every possible percentage of identity. The representative sequences were 240 bases long after removing one base at every clustering level until we reached one base similarly among ASVs. This resulted in a range from 100% identity to 0.004% identity. It is essential to note that the ITS region seems to fail as a phylogenetic tool as the region is polymorphic, short, and presents high variability [35, 43, 44]. In fact, within a single strain, considerable sequence divergence between copies of the ITS1 region has been documented [45]. Our intent here was to indicate how certain classified ASVs might relate to others, giving or reducing the weight of the certainty on the taxonomic classifications.

All the ASVs that were classified as *Candida* to genus level are compared along with two strains of *Candida albicans* (NCBI accession number:MT166273.1, MN960653.1) [46, 47] and one strain *[Candida] nivariensis* (NCBI accession number:KY102231.1) [48]. The classification of the ASVs against three databases and their trust score, and the identity value from a pairwise comparison for each ASV were generated from the R package Bio3d [49] after [50].

## Results

In this study, we obtained a total of 3,096,321 and 2,609,027 non-chimeric reads for the bacterial (16 S rRNA) and fungal (ITS) datasets, respectively. The bacterial dataset had a minimum of 77,113 reads, a maximum of 145,189 reads, and a mean of 103,211 reads per scat sample. The fungal dataset exhibited a minimum of 6,734 reads, a maximum of 166,246 reads, and a mean of 86,968 reads per scat sample.

### Alpha diversity

**Fig. 2:**
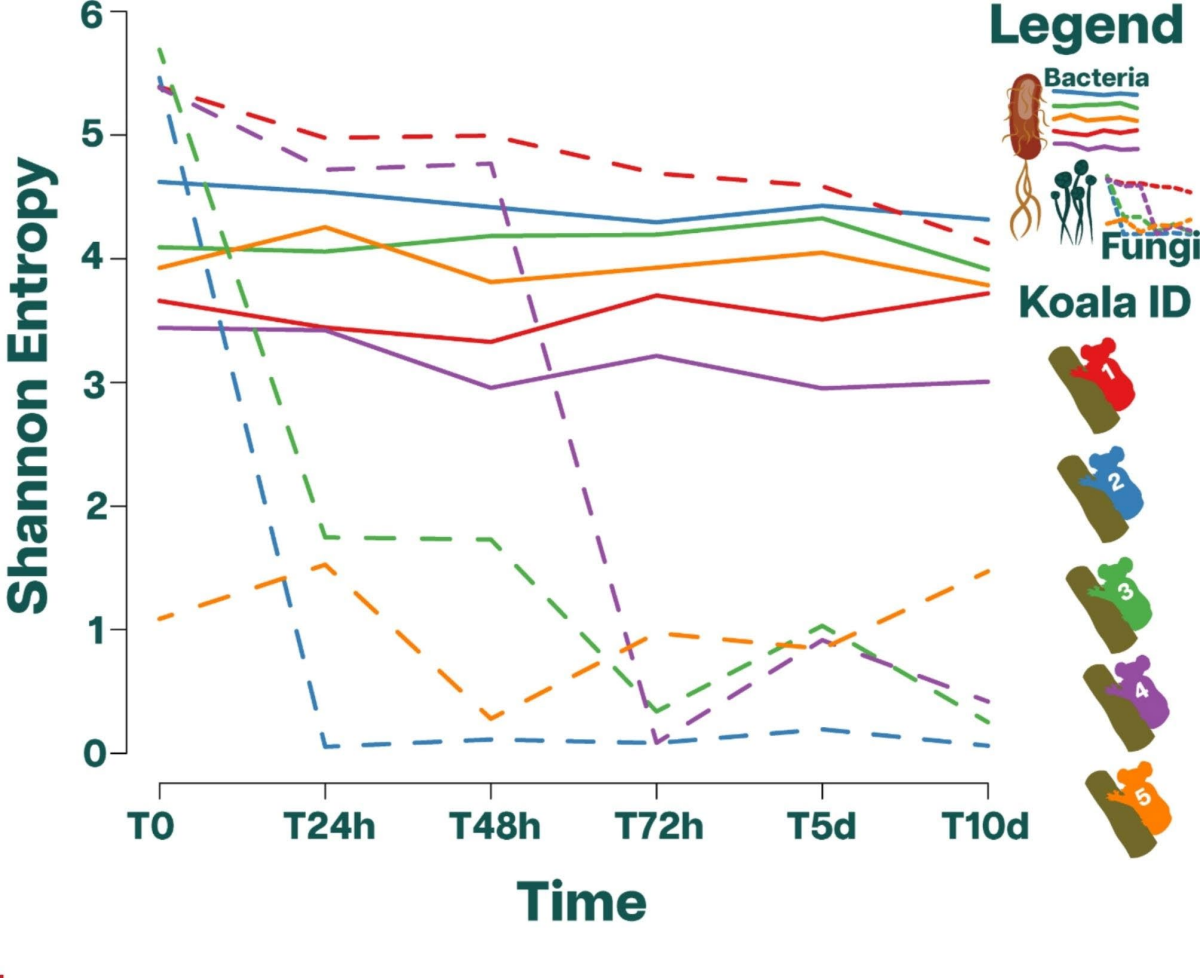
Comparisons of alpha diversity between koalas. The relationship between time (scat aging) and Shannon entropy coefficient for the scat samples taken from five different koalas. Each koala id (color-coded in the plot) is represented by six time-points ranging from 0 to 10 days. The Shannon Entropy coefficient was determined at each point in time. In the combined plot, dashed lines represent Fungi (ITS1 and ITS2), and solid lines represent bacteria (16s V3 and V4)

Scat aging impacted the bacterial and fungal alpha diversity differently. The bacterial alpha diversity was found to persist and remain stable through time (Table 2; Fig. 2A). A Friedman test confirmed this stability (χ2 = 4.66, p = 0.45) with post-hoc comparisons showing no significant differences between any of the time points. These results indicate that scat aging for 10 days had little to no impact on the bacterial alpha diversity in our samples. In contrast, the alpha diversity of fungi exhibited significant differences over time, indicating that it did not remain constant (χ2 = 11.29, p = 0.046). Post-hoc comparisons revealed significant differences between the following time points: T0-T72h, T0-T5d, and T0-T10d. Further analysis revealed the following behaviour: diversity inter-koala difference at T0 were minimalexcept for Koala 5. In two of the five koalas sampled for scats, alpha diversity remained stable through the scat aging process. Koala 1 exhibited a consistently high alpha diversity profile, while Koala 5 consistently displayed a low profile (Table 2; Fig. 2). In contrast to the bacterial alpha diversity, scat aging significantly impacted the fungal alpha diversity in three of the five koalas. Over a period of 24 to 72 h, these koalas experienced notable changes, their alpha diversity shifting from the highest to the lowest levels (Fig. 2B). Thus, these data suggest a potential inconsistency: while bacterial alpha diversity demonstrated stability throughout the aging experiment, the fungal counterpart seemed to display a higher degree of variability.

**Table 2:** Summary of alpha diversity metrics at time zero (T0) and after T0 for bacteria (16s V3 and V4) and fungi (ITS1 and ITS2) in koala scat samples: This table presents the minimum, maximum, mean, and standard deviation (SD) values for alpha diversity metrics, including richness, evenness, and Shannon index, at time zero (T0) and after T0 across all koalas. The T0 values represent averages across all koalas at time zero, while the values after T0 represent averages across all time points and koalas beyond time zero

Alpha Diversity: T0								
Index	Bacteria (16s V3 and V4)				Fungi (ITS1 and ITS2)			
	Min	max	mean	SD	min	max	mean	SD
Richness	159	221	190	27.60	74	169	118	39.50
Evenness	0.47	0.61	0.67	0.05	0.76	0.85	0.79	0.04
Shannon	3.40	4.60	3.90	0.46	1.09	5.70	4.60	1.96
**Alpha Diversity After T0**								
Richness	124	239	178	31	9	133	46.32	41
Evenness	0.40	0.60	0.51	0.053	0.76	0.30	0.31	0.28
Shannon	2.90	4.20	3.80	0.48	0.05	4.99	1.80	1.91

### Beta diversity

**Fig. 3:**
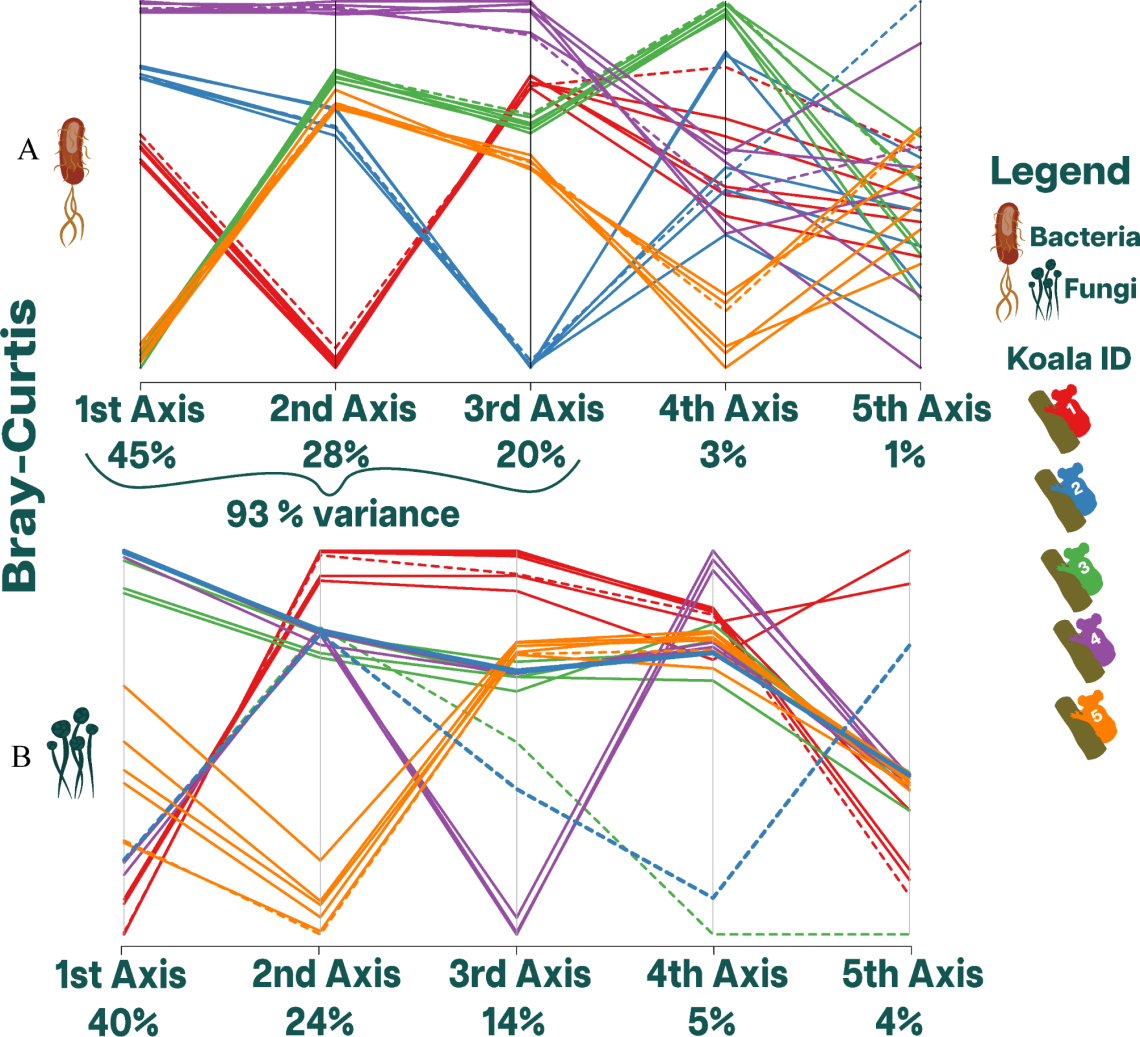
Comparison of beta diversity between koalas. Bray Curtis dissimilarity principal coordinates analysis represented by a parallel coordinates plot. The scat samples taken from five koalas are represented by six points in time from 0 to 10 days. Therefore, each color denotes a koala id (color-coded in the plot) with six lines representing those time points. The first five principal axes were plotted, and the total amount of variance they account for was printed on the x-axis. Plot (**A**) accounts for bacteria (V3 and V4), and plot (**B**) accounts for Fungi (ITS1 and ITS2). The dashed lines represent the T0 time point, while the solid lines represent the other time points

Here, we aimed to identify where the largest variance was found, whether between time points (intra-koala differences) or between individuals (inter-koala differences). If the intra-koala differences are smaller than inter-koala differences, the variance introduced by time is negligible. If an axis satisfies this rule, we determine that the amount of variance represented by it is not skewed by time. We verified this rule in each one of the first five principal coordinate axes, adding the variance that each axis represents only if the rule was satisfied, and concluded that the added amount of variance withstands the effect of time (scat ageing). For example, in bacteria using Bray-Curtis, the first five axes of the principal coordinates captured 96% of the variance. However, the analysis yielded that the intra-koala difrences were smaller than inter-koala difrences for the first three axes. These three axes represented the added total of 93% of the variance. Thus, from an abundance (Bray-Curtis) perspective, approximately 93% of the variance in bacterial load measured ASVs withstand time (Fig. 3A).

For fungi, the first five axes explained 87% of the variance. Koalas 2, 3 and 4 started at T0 with a more similar fungal Bray-Curtis distance which takes abundance into account (Supplementary Fig. 3A). The most distinct individuals were Koala 1 and Koala 5, as they had extremely different fungal compositions from each other and the rest of the individuals. When we included all the time points measured for all koalas, the individual differences in time became extreme, especially for Koalas 2, 3 and 4. Koala 5 had a less extreme yet considerable difference, while Koala 1 was an outlier with the least differences between time points. Even though Koalas 2, 3 and 4 started with a similar composition at T0, the differences between them were clear (Supplementary Fig. 3A). However, at T10d, these three individuals clustered from axis 1 to axis 5 (Supplementary Fig. 3B). Thus, for 91% of the variance, the Bray-Curtis distance differences between Koala 2, 3, and 4 at T10d were approximately 0 (supplementary Fig. 3B). In fungi, the extreme effects of time removed the differences between individuals; this can be seen in time points 72 h, 5d, and 10d (Supplementary Fig. 3B). However, in two individuals, the effects of time were not as extreme. For Koala 5, the influence of time can be discerned from the first two axes (Fig. 3B). Finally, Koala 1 was an outlier as time had a substantially smaller effect compared to the other individuals. As shown in Fig. 3B, Koala 1 maintained the trend from axis 1 to axis 4. Furthermore, the maximum intra-koala difference observed in Koala 1 between two distinct time points is 4.6 times smaller than the average maximum difference observed in inter-koala comparisons. Thus, with the exception of Koala 1, time substantially affected the abundance of the fungal composition measured as a function of Bray-Curtis distance (Fig. 3B). Jaccard distance showed a similar profile to Bray-Curtis but did not explain as much of the variance given; it did not include abundance measures. The results and plots of Jaccard distance can be found in the Supplementary Material.

#### Effect of time on bacterial beta diversity metrics

**Fig. 4:**
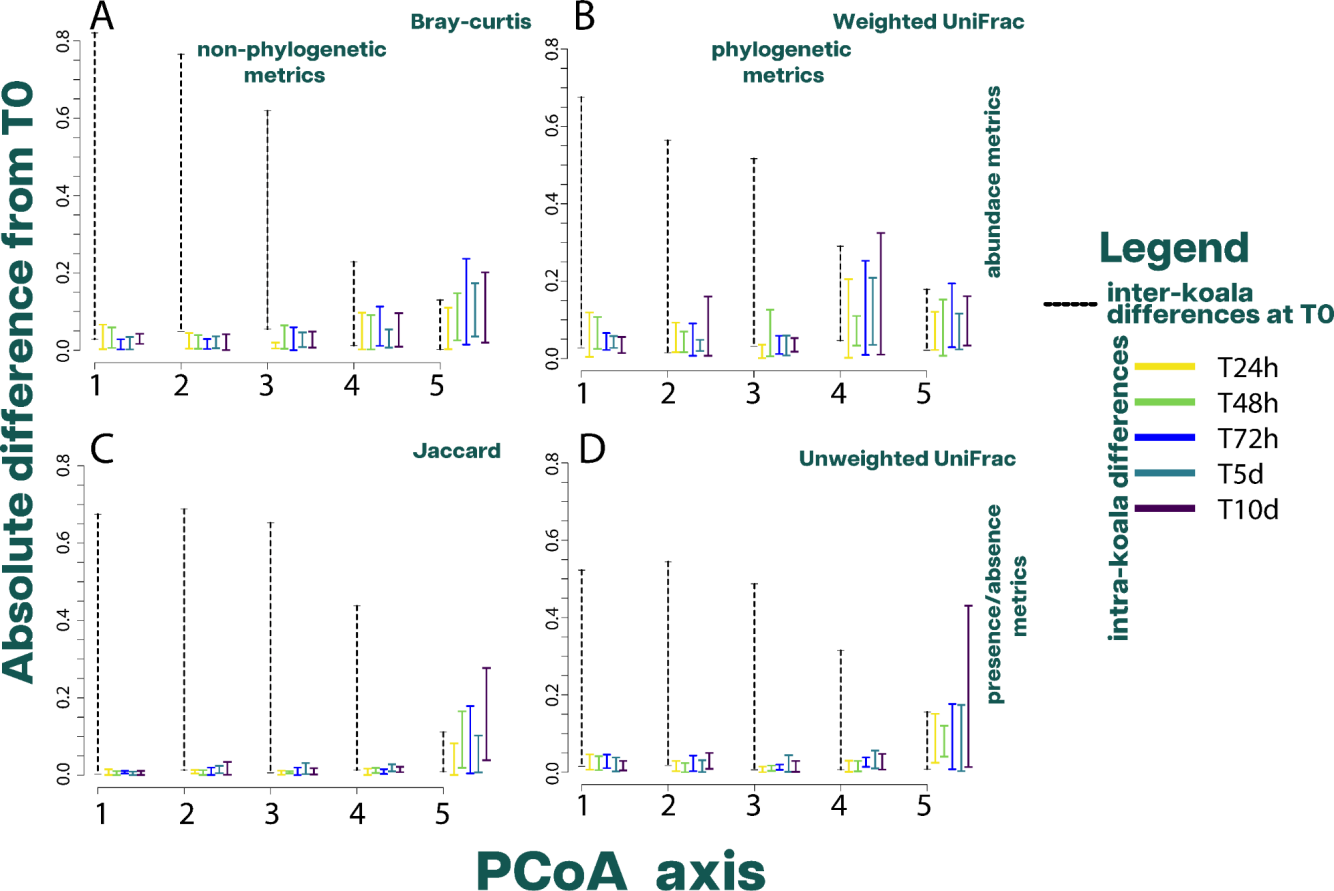
The effect of scat aging on bacterial beta diversity metrics. The graph compares the range of absolute intra-koala differences between T0 and each time point (T24h to T10d) with the range of absolute inter-koala differences at T0 for the first five PCoA axes across four different beta diversity metrics. (**A**) Bray-Curtis (**B**) Weighted UniFrac (**C**) Jaccard (**D**) Unweighted UniFrac

In general, for the first three axes, which account for the majority of the variance in all beta diversity metrics, the inter-koala absolute difference at T0 was greater than the intra-koala absolute difference from T0 to the remaining time points (Fig. 4). This finding was consistent with the Friedman test, as time was insignificant in any beta diversity metric for the first three axes. However, from the fourth axis onwards in the metrics that relied on abundance (Bray-Curtis and Weighted UniFrac), the intra-koala differences were comparable to or larger than the inter-koala differences (Fig. 4A and B). For the fourth axis, the Friedman test was only significant for the Jaccard distance (χ2 = 13.11, p = 0.022). The post hoc analysis reveals that only T5d had a significant difference from T0, with a difference of 11 ranks (χ2 = 13.11, p = 0.026). For the fifth axis, all beta diversity metrics presented equal or greater intra-koala differences than inter-koala differences (Fig. 4). On this axis, the Friedman test was only significant for Bray-Curtis (χ2 = 13.23, p = 0.021). The post hoc analysis showed that times greater than T72h had a significant difference from T0, which ranged from 14 to 16 ranks, with chi-squared values of 13.23 and p-values between 0.002 and 0.005.

**Fig. 5:**
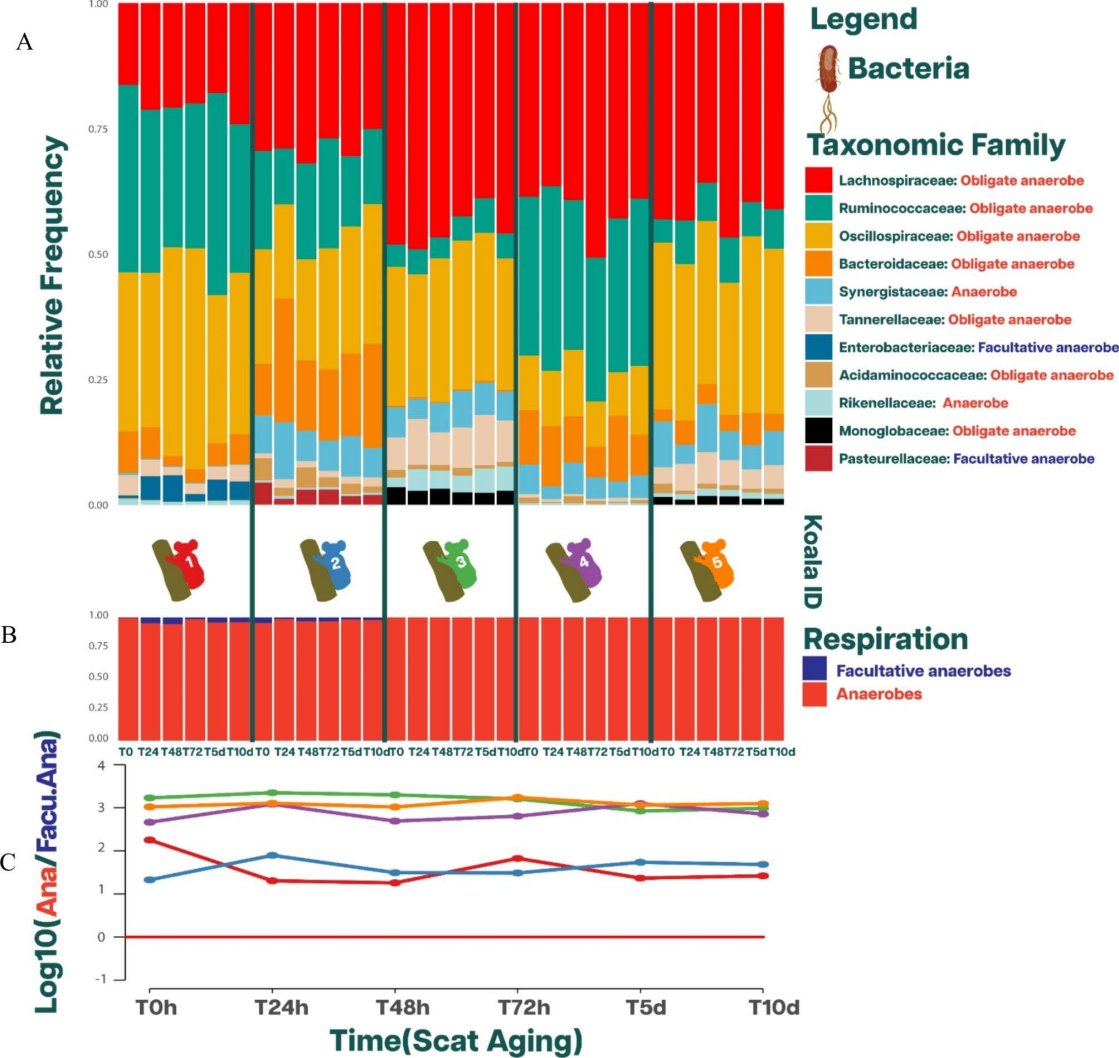
Impact of oxygen on bacterial taxa through the scat aging process (**A**) Relative frequency of the 11 most abundant bacterial taxa at a family level during the scat aging process. Each bacterial family has its respiration profile. (**B**) Relative frequency at taxonomic family level, group by its respiration profile through the scat aging process. (**C**) Logarithmic ratio of anaerobic to facultatively anaerobic bacteria. The red line marks the threshold below which facultatively anaerobic would be more abundant than anaerobic bacteria, which does not occur through the scat aging process

While the inter-koala differences in bacterial community remain relatively the same when comparing non-phylogenetic metrics with their phylogenetic counterparts (Jaccard vs. Unweighted UniFrac and Bray-Curtis vs. Weighted UniFrac), the intra-koala differences increased when phylogeny was considered in all axes (Figs. 4 and 5). Then, when we compared presence/absence of bacteria with abundance beta diversity metrics, the abundance metrics presented higher intra-koala differences than presence/absence metrics (Figs. 4 and 5). In fact, the gradient in which the beta diversity metrics increase in intra-koala differences is shown in Supplementary Fig. 5, demonstrating that not all beta diversity metrics respond the same to the effects of time on bacteria.

**Fig. 6:**
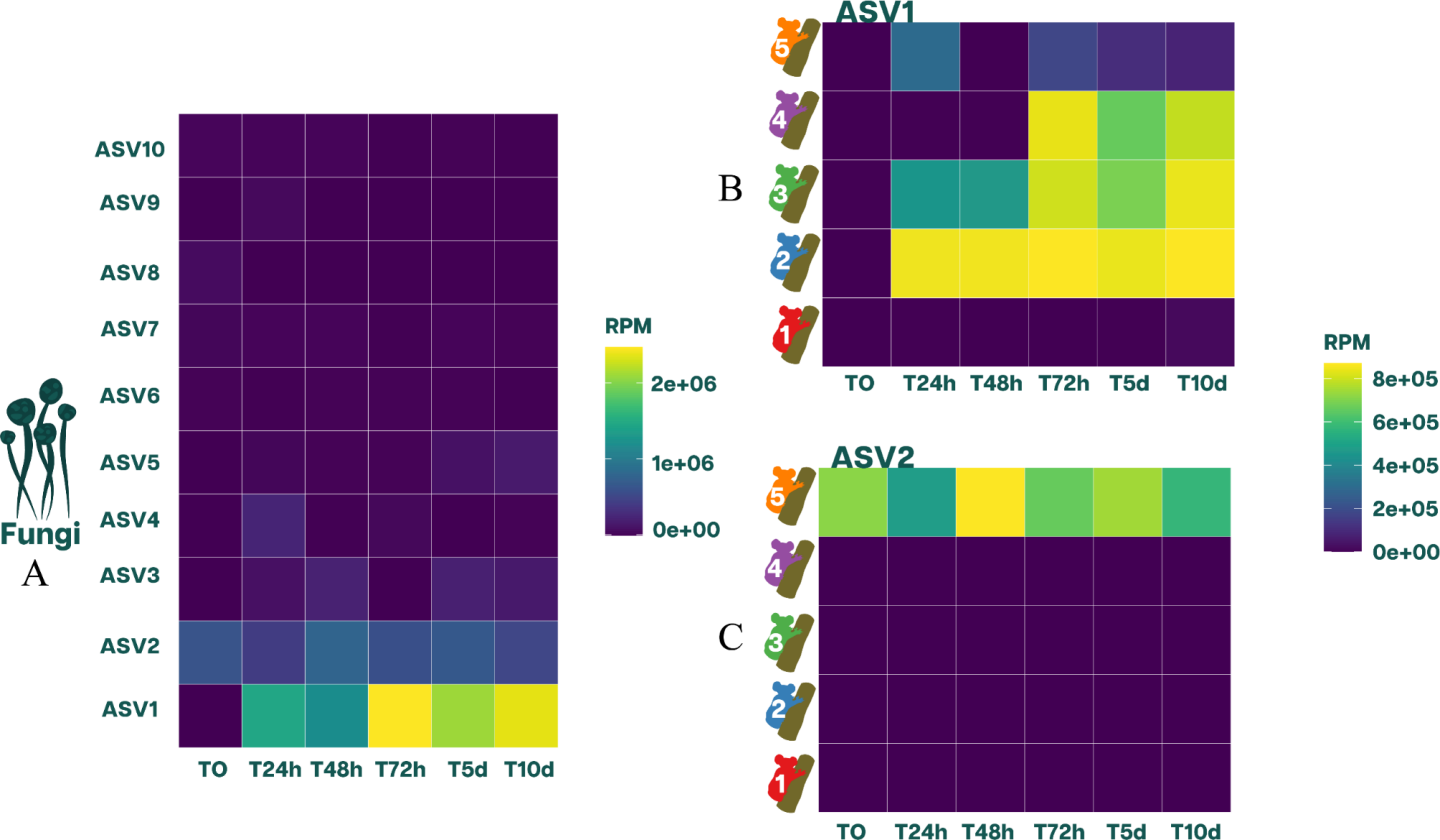
The most abundant ASVs for Fungi (ITS1 and ITS2) across time (scat aging). (**A**) The 10 most abundant ASVs for each of the six points in time. These were calculated as the sum of the Reads Per Million (RPM) for the scat samples of the five koalas for each specific time point. (**B**) The most abundant ASV1 in RPM for each koala per time point. (**C**) The second most abundant ASV2 in RPM for each koala per time point

We did not observe a depletion in obligate anaerobes nor an increase in facultative anaerobes (Fig. 5). In fact, the logarithmic ratio has a positive number through the scat aging process even after 10 days (Fig. 6B and C). In other words, anaerobic bacteria were present in larger proportions than facultative anaerobes. Furthermore, we did not observe any obligate aerobes or aerobic bacterial taxa in the pool of samples at any point in time.

### ASVs relative abundance and their journey through time

By establishing how ASVs changed through time, we can better explain the patterns found in the alpha and beta diversity results. A total of 1,054 ASVs were identified across the entire set of scat samples, representing a total of 2,609,027 reads. Of this total, 7.6% of the fungal reads were found in T0 across all koalas, while T24h and T48h had 16.2% and 14.1% of the fungal reads, respectively. After these time points, we observed an increase in the percentage of fungal reads. The fungal reads for T72h to T10d represented 20.1–21.1% of the total reads. The most substantial increase in the percentage of fungal reads is observed at T72h. This significant increase is important as it highlights a potential shift in the fungal community composition over time.

Given this context, we now turn our attention to the specific ASVs contributing most significantly to these fungal reads. ASV1 and ASV2 represent 58.8% and 21.9% of the total fungal reads, respectively. These high percentages demonstrate the dominance of ASV1 and ASV2 compared to the rest of the ASVs in terms of relative abundance. This observation is consistent with Fig. 6A, which clearly shows the insignificance of other ASVs relative to ASV1 and ASV2.

Even though ASV1 was present at T0 in the pool of samples, it only represented 0.059% of the total fungal reads at that time point. After 24 h, it surged to represent 50.9% of the reads. Then it decreased to 40.1% of the reads at T48h, to dramatically increase to 79% of the reads at T72h. Finally, it slightly decreased yet remained above 66.4% for T5d and T10d (Fig. 6A). ASV1 heavily invaded three out of the five individuals represented by the scat samples after T0 (Fig. 6B). For instance, ASV1 represents 99.5% of the fungal reads of Koala 2, 96.7% of Koala 3, and 94.1% of Koala 4 at T10d, making the rest of ASVs virtually 0 at that point in time.

The second most abundant ASV (ASV2) was only present in Koala 5, representing 21.9% of the total fungal reads (Fig. 6C). In contrast to ASV1, this fungus was highly abundant at T0 and represented 46.1% of the fungal reads at that time point. This fungus remained stable through time (Fig. 6C), explaining why Koala 5 had a Shannon index around 1 from T0 to T10d (Fig. 2B).

Finally, we argue that only the ASVs present at T0 represent the true composition and relative abundance of fungi in koala scats sampled as measurable changes were observed from the second time-point onwards. Of the 1,054 fungal ASVs found in the pooled sample, 519 were present at T0, representing 11.6% of the total fungal reads. Of the ASVs present at T0, 81.6% were exclusive to an individual, 13.0% were present in two individuals, 2.7% were present in three individuals, 1.1% were present in four individuals, and 1.3% were present in all 5 individuals.

### Fungal taxonomy

**Fig. 7:**
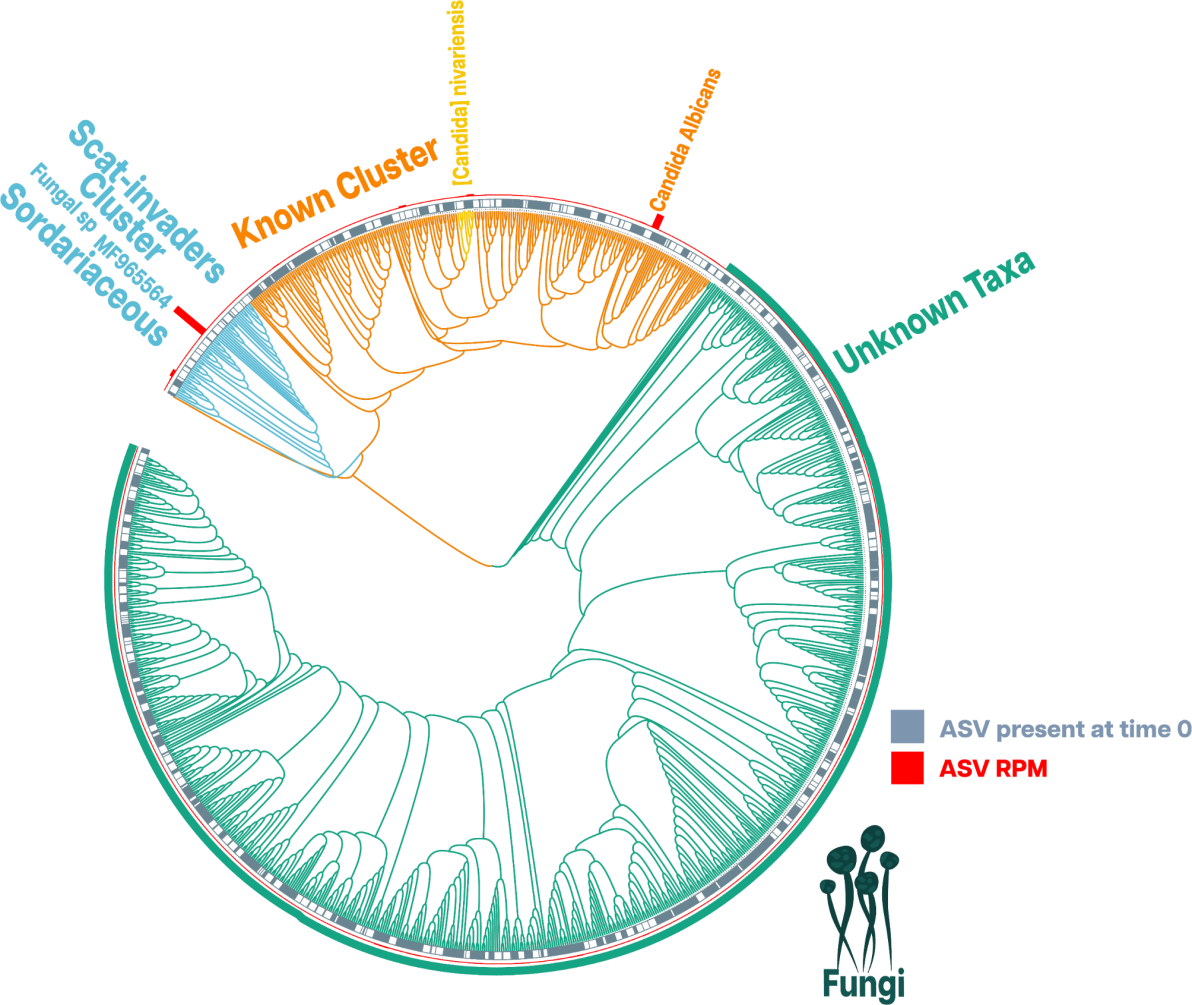
A cluster-based visualization of the 1054 Fungi (ITS1 and ITS2) ASVs found in the pool of stool samples. Grey squares represent the ASVs present at time 0, while white squares represent the ASVs not present at time 0. The red bar plots represent the relative abundance in RPM for each ASV. Two significant clusters were identified in this figure. These are represented by branch colour: *Scat-invaders* cluster in blue, and the *Known* cluster in orange. The branches in green colour represent all the ASVs that did not match any taxonomic identification that came from the Unknown clusters (*Big-Unknown, Small-Unknown*, 3, 5 and 8) here represented as one cluster, while branches with a different colour were loosely assigned to a taxonomic group

Despite using several databases and taxonomic assignment tools as described in the methods, the success rate of classifying the fungal ASVs was very low. Of the 1,054 fungal ASVs (ITS1 and ITS2) present across all scat samples, only 287 (27.2%) received a taxonomic rank, while the remaining 767 ASVs were completely unassigned (Fig. 7). We performed cluster analyses in an attempt to resolve this issue and better understand the taxonomic diversity present in koala scat samples. For instance, applying the threshold of 1 nucleotide similarity between ASVs resulted in nine clusters. These nine clusters persisted as we increased the identity coverage up to 35% similarity between ASVs. Cluster 1 included 731 ASVs, all entirely taxonomically unassigned. From here on, we will refer to this cluster as “*Big-Unknown*”. Cluster 2 included 235 ASVs, and most of them received at least a taxonomic rank higher or equal to Phylum. From here on, we will refer to this cluster as the “*Known*”. Clusters 3, 5, 8, and 9 included between one to three ASVs, except for Cluster 9, all the ASVs remained taxonomically unassigned. Cluster 4 included 31 taxonomically unassigned ASVs. From now on, we will refer to this cluster as “*Small-Unknown*”. Cluster 6 included 49 ASVs, with most of them receiving at least a taxonomic rank higher or equal to genus level. Many ASVs in this cluster shared the similarity that most of them were not present at T0 (Fig. 7). For this reason, we will refer to this cluster as the “*Scat-Invaders*”.

It is important to note that there was not a single nucleotide position that could be aligned between these nine clusters. However, within them, the nucleotide similarity to the parent ASV was much higher though, varied greatly. In terms of percentage identity within clusters, *Scat-Invaders* were the most similar. The percentage identity ranged from 65 to 99.2%, with a mean of 80.6% and an SD of 9.3%. The least similar cluster, the *Known* cluster, had a percentage identity that ranged from 36.2 to 99.6%, with a mean of 59.9% and an SD of 13.8%.

Of those ASVs we could assign a taxonomic rank, defining characteristics from this information was a difficult task. However, there were some ASVs for which the taxonomic classification was more informative. In particular, ASV1, the most abundant ASV in the pool of scat samples, was classified with high confidence using the BLAST search in NCBI; ASV1 matched the ITS1 region of Fungal sp. accession number MF965564.1 [51] with an identity and query coverage of 100% for both criteria. Furthermore, this fungal species might be closely related to a *Sordaria alcina* isolate (accession number EU551182.1) [52], as the identity between the two is 99%. We also classified all ASVs against UNITE and RefSeq. ASV1 was classified at the family level to *Sordariaceae* by both databases. This classification provided a good indication, as all the ASVs from the *Scat-Invaders* were classified at the family level as *Sordariaceae*, with 68.7% of them further classified at genus level to *Neurospora*. We, however, found a different pattern when we classified the *Scat-Invaders* cluster using the BLAST search engine in NCBI with 24% ASVs classified as *Sordaria*, while others were classified as Fungal sp. ASV1 clustered with nine other ASVs the BLAST search classified as Fungal sp most probably *Sordaria alcina*. It was only when we utilised a lower identity coverage than 98% when the ASVs classified by the BLAST search classified as *Sordaria* at the genus level were included in this cluster group (Fig. 7).

**Fig. 8:**
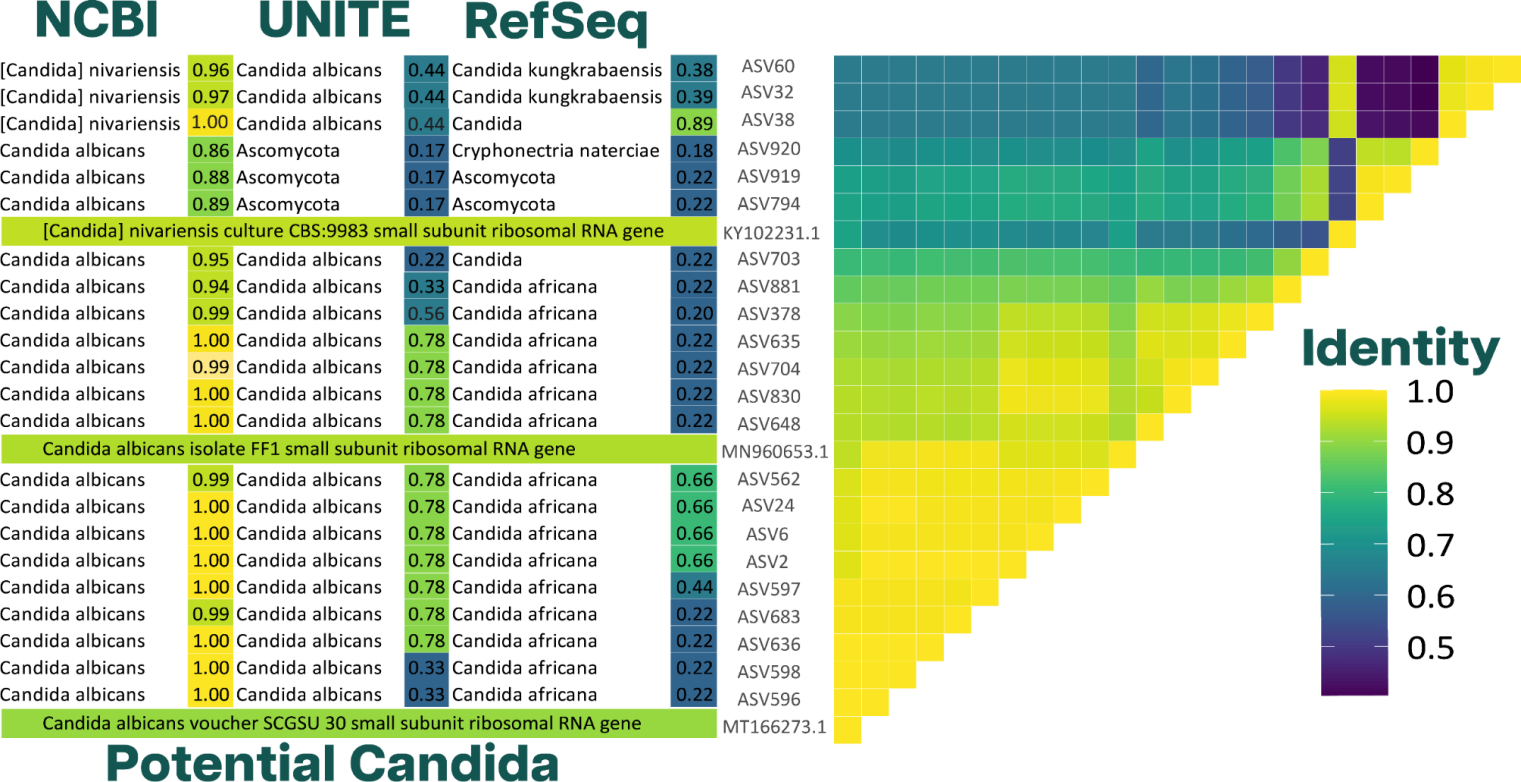
Potential *Candida*: Heatmap represents all the ASVs that were classified as Candida to genus level compared with two strains of *Candida albicans* and one strain [*Candida*] *nivariensis*.On the left, the figure displays the classification of the ASVs against three databases and their trust score. On the right, The identity value from a pairwise comparison for each ASV after aligning their sequences

The second most abundant fungus in the pool of scat samples, ASV2, was also classified at the species level with high certainty. Using BLAST search, this ASV had best matching hits exclusively to different strains of *Candida albicans* with an identity of 100% and a query coverage of minimum 97%. We also classified 22 ASVs from the *Known* cluster at the genus level to *Candida*, except for three (ASVs 32,38 and 60), all of them were classified as *Candida albicans*. Figure 8 displays an identity pairwise comparison of these 22 ASVs, and their classification against three databases. It is important to note that the ASVs classified as *Candida albicans* were only present in Koala 5. While the remaining three (ASVs 32,38, and 60) classified as [*Candida*] *Nivariensis*, were only present in Koala 1. In terms of sequence similarity, the ASVs identified as *Candida Albicans* are quite distinct from the ASVs identified as [*Candida*] *Nivariensis* (Figs. 8 and 9).

## Discussion

### The effects of scat aging on bacterial community stability

In this study, the main premise is that if the intra-koala differences in diversity metrics are smaller than the inter-koala differences, we can conclude that the effects of time do not significantly skew the factors driving the composition and abundance of ASVs. From an alpha and beta diversity perspective, bacteria seem to be stable through the aging process. The results demonstrated a remarkable consistency across all individuals, as all beta diversity metrics were able to distinguish individual koalas throughout the scat aging experiment. This indicates that, for the majority of the variance observed, the intra-koala differences did not substantially impact the inter-koala differences. As a result, we were able to identify and differentiate individual koalas regardless of the specific time point in the experiment. In fact, the Freidman test shows that time did not have significant effect in the first three axes in all beta diversity metrics, which account for 68% or more of the variance for each metric being evaluated. Furthermore, all beta diversity plots separate individuals and form distinguished clusters in the first three axes. We note that from life history information, the factor driving these clusters appears to be location, which is supported by the current literature [10].

In our investigation, we measured the impact of time on the beta diversity metrics of bacterial communities. The temporal variance was observed to influence beta diversity metrics distinctively, contingent on whether the metrics consider the presence or absence of ASVs, their relative abundances, or their phylogenetic relationships (Fig. 4 and Supplementary Fig. 5).

Interestingly, the Jaccard metric, which accounts for the presence or absence of ASVs, displayed minimal response to scat aging (Supplementary Fig. 4A). This result underscores a potential resilience in the bacterial community across time, with the same ASVs consistently present despite the passage of time. Conversely, the Bray-Curtis metric, which factors in the relative abundances of ASVs, revealed a slight but not significant response to aging. This suggests that while the diversity of ASVs remains generally unchanged, there is a subtle shift in their individual contributions to the overall community dynamics as time progresses. Furthermore, we noted nuanced responses from the unweighted and weighted UniFrac metrics that shed light on the influence of phylogenetic relationships within the bacterial community. Despite scat aging, the unweighted UniFrac metric - encapsulating changes in the presence or absence of ASVs whilst accounting for their phylogenetic relationships - remained largely stable, suggesting a robust phylogenetic structure within the bacterial community resistant to the passage of time (Supplementary Fig. 2A). However, the weighted UniFrac metric, which incorporates both relative abundance changes and phylogenetic relationships, exhibited a more pronounced but not significant response to aging (Supplementary Fig. 2B). This outcome suggests that closely related ASVs exhibited similar responses to the aging process in terms of changes in their abundance.

Previous studies reported anaerobic bacteria decreased, whereas facultatively anaerobic and aerobic bacteria increased during the scat aging process [26–29]. However, the rate in which these occurred vary, suggesting that the stability of scat microbiota is dependent on the species of wildlife due to the susceptibility of scats to the aerobic environment [26, 27, 29]. In fact, Menke et al., [26] suggested that using the core of the scat pellet minimizes the amount of exogenous bacterial contamination and increases the chance to sample a better-preserved scat region. We were able to corroborate this since we did not observe a reduction in obligate anaerobic bacteria nor an increase in facultative anaerobes even after 10 days. This is likely attributed to the fact that during the DNA extraction process, we targeted the core of the sample, while other studies broke the sample in three equal portions without avoiding the external surface [29]. Therefore, it is important to assess the integrity of faecal samples upon collection in the wild to ensure that the core has not been exposed to air and to target this portion of the scat for DNA extraction.

### The effects of scat aging on fungal community stability

Fungal composition and abundance measured at T0 did not form distinct clusters between individuals such as those observed in bacteria (Fig. 3). Moreover, the scat aging process greatly affected the fungal composition and abundance of the scat samples, as alpha diversity had an overall decrease. In terms of beta diversity, the dissimilarities between samples decreased over time due to the scat aging experiment. However, not all individuals respond equally to the scat aging process; two out of three samples had significantly different fungi compositions from T0. Only Koala 1 exhibited somewhat stable alpha and beta diversity through time. A possible explanation for this may be due to its distinct fungal composition compared to the rest of the individuals that may have been shaped by the exposure to antibiotics. Perhaps this particular composition allowed scat samples from Koala 1 to maintain a stable alpha and beta diversity over time.

### ASV1 and the *Scat-Invaders*

Within the fungal composition, the aging effect was potent enough that after 72 h, most of the samples had no differences between individuals from an abundance perspective. The ASV relative abundance analysis revealed the primary driver of the change in abundance; ASV1 was later identified as a recently described fungal species found in Australia [51], which might be closely related to *Sordaria alcina*. This species had been isolated from koala scats [52]. Most ASVs present in the Scat-Invaders cluster were classified into the family Sordariaceae. However, there was a lot of divergence between the classifications at a genus level between databases. Fortunately, the distribution of this taxonomic family had been studied extensively [53, 54]. *Sordariaceous* were typically found in humid tropical and subtropical regions [53, 55]. However, *Sordaria* was widely known for being a coprophilous genus, only being found in scats [53, 56, 57]. In contrast, Neurospora was known for the opposite, generally found in soil samples but absent in scats [53, 58]. Thus, the Scat-Invaders cluster was most likely composed of fungi related to this new fungal species and the Sordaria genus.

One possible way of dealing with the effects of aging when trying to uncover the fungal composition and abundance of scat samples in tropical and subtropical regions is to filter out these taxa. However, we concluded that from 72 h of scat aging, the fungal communities of the scat sample may be compromised not only from an abundance perspective but also from a presence-absence perspective, producing a sample that does not accurately represent the fungal communities present at T0. Thus, it is highly recommended not to use samples older than 72 h.

### Exploring the complexities of fungal classification

The taxonomic classification was challenging despite encountering a group of fungi that appear to colonise scats. As shown in the results, most fungal ASVs were completely unassigned, and many that received a taxonomic rank presented high uncertainty. It is a fact that the study of the fungi sub-kingdom is underdeveloped compared to bacteria. It has been estimated that only 1% of fungal species have a reference in a database [59]. Furthermore, there is a lack of curation on fungal databases [8, 59, 60]. The ITS region is one of the most common metabarcodes used in fungal diversity studies [7]. However, it might not be the best region to amplify, due to its highly polymorphic nature. For example, some Ascomycota lack interspecific variation, while other fungal species vary heavily between related species [35, 61–67]. This somewhat explains the poor characterization of fungal communities in general and the inability to perform a robust phylogenetic analysis, which is the current standard for diversity metrics, as they account for the degree of divergence between sequences [68–70]. Perhaps, the use of long reads for amplicon sequencing might address the problems that the ITS region presents. Long-read sequencing technologies are not nearly as constrained by the length of region used for the metabarcode and could target amplicons severalfold larger than what current short-read methods can manage[35, 71]. Such approaches might be able to provide a more robust view on the phylogeny of the fungal composition of the scat sample given a much larger array of nucleotide positions to compare.

### *Candida Albicans*: the common mammalian pathogen

The second most abundant ASV was identified as *Candida albicans.* This yeast represented approximately 20% of the total fungal reads. Nevertheless, this yeast was restricted to only one individual (Koala 5). The genus *Candida* includes approximately 160 species. However, this genus is currently under revision [48]. *C. albicans* is arguably the most clinically significant species of *Candida* [7]. This yeast tends to cause infections primarily after the host has undergone antibiotic treatment [7]. It was surprising that this fungal yeast was not observed in Koala 1 given this individual was recently administered antibiotics to treat *Chlamydia*. Instead, we found ASVs that seem more closely related to *[Candida] nivariensis*. From our analysis, these two species appear quite different. Though, this divergence may be due to the choice of target region. Curiously, we dug into the veterinary history of Koala 5, and it seems that he always had been a healthy subject, without any history of *Chlamydia* or any known antibiotic administration.

## Conclusions

While time had a small effect on the bacterial composition of the scat samples, we show that more robust beta diversity metrics that consider phylogeny are better able to capture and identify how time is affecting the microecology of the samples. In fungi, the effect of time was indisputable, as we encounter a whole fungal community that invades and colonizes scats changing its composition and abundance, which is unsurprising, given the fungi kingdom is known as one of the most diverse and abundant decomposers in the environment [8]. However, the exploration of the scat samples’ fungal community was constrained due to the early stage of the fungal metabarcoding databases development.

To further the scope of conservation metagenomics as a tool to monitor endangered species in the wild, it is essential to first understand the factors that might skew the sample being collected. A non-invasive metagenomic sample will be subject to environmental degradation as a function of time. Thus, it is fundamental to understand its effects and safeguard the sample’s quality. Our study suggests that field-collected scat samples can indeed serve as valuable data sources in conservation studies. We found that in koalas, most of the variance in bacterial diversity between individuals was maintained over time, despite the aging process of scats. This key finding enables future exploration of conservation strategies. It provides a foundation for potential development of monitoring programs, streamlined translocation efforts, and the discovery of health biomarkers. Therefore, our study’s results could pave the way for a deeper understanding of individual bacterial diversity, a factor that could significantly influence decision-making processes in future conservation research. However, it’s essential to bear in mind that these findings may vary for different species. As each animal’s scat undergoes unique changes in microbial composition when shifting from an anoxic to an aerobic environment, it is crucial that these aging experiments be conducted for each species under consideration. Hence, further research is needed to establish the robustness and applicability of our findings across different taxa.

## Electronic supplementary material

Below is the link to the electronic supplementary material.

Additional file 1: Contains the following supplementary results, figures, and tables: Supplementary Results: Detailed discussion and analysis of the Jaccard dissimilarity (PCoA) results. The discussion focuses on the presence/absence of ASVs in bacteria and fungi over time and how these affect the gut microbiome. Figure S1: Picture of Scat aging setup. Figure S2: Phylogenetic beta diversity plots for bacteria. Figure S3: Effect of time after 72 hrs of scat aging in fungi. Figure S4: Comparison of beta diversity between koalas. Figure S5: The effect of scat aging on bacterial beta diversity metrics. Table S1: Bacteria Quality control metrics. Table S2: Fungi Quality control metrics. Table S3: Respiration profile of 27 most abundant taxonomic families for bacteria.

## Data Availability

Raw sequence reads are deposited in the NCBI SRA database under BioProject PRJNA891490 https://dataview.ncbi.nlm.nih.gov/object/PRJNA891490?reviewer=8f4jem4pns9d1ulbp0cgctpvsj. Custom code used for this study is available from the figshare repository 10.6084/m9.figshare.21357807.
