# Supplementary material for: Influence of scat ageing on the gut microbiome: how old is too old?
Source: BMC Genomics. 2023 Jul 31;24:427. doi: 10.1186/s12864-023-09520-0 (PMC10388479; doi:10.1186/s12864-023-09520-0)
Supplement: Supplementary file 1 — Additional file 1: Contains the following supplementary results, figures, and tables: Supplementary Results: Detailed discussion and analysis of the Jaccard dissimilarity (PCoA) results. The discussion focuses on the presence/absence of ASVs in bacteria and fungi over time and how these affect the gut microbiome. Figure S1: Picture of Scat aging setup. Figure S2: Phylogenetic beta diversity plots for bacteria. Figure S3: Effect of time after 72 hrs of scat aging in fungi. Figure S4: Comparison of beta diversity between koalas. Figure S5: The effect of scat aging on bacterial beta diversity metrics. Table S1: Bacteria Quality control metrics. Table S2: Fungi Quality control metrics. Table S3: Respiration profile of 27 most abundant taxonomic families for bacteria. [file 12864_2023_9520_MOESM1_ESM.docx]

**INfluence of scat ageing on the gut microbiome: how old is too old?**

Alejandro Oliveros^1,3^, Julien Terraube^2,3^, Alexis L. Levengood^3^, Daniel Powell^3,4^ and Céline H. Frère^1,3,4^

**Supplementary Results**

Jaccard dissimilarity (PCoA)

Jaccard distance matrix only accounts for pair-wise comparisons of presence/absence of ASVs. In bacteria, the first five axes of the principal coordinates captured 70% of the variance. Supplementary Figure 4A shows how at T0 individuals cluster distinctly for the first four axes, suggesting that these groups share the same ASVs but are distinct between groups. When we included all the time points from T0 to T10d measured for all koalas, the clustering through principal coordinates axes was maintained as the differences in time within individuals are far less than between clusters and individuals. From a presence-absence perspective, approximately 69% of the variance in bacterial load measured ASVs withstand time (scat aging).

In contrast, the presence-absence of fungal composition measured as a function of Jaccard distance when using all time points and accounting for all individuals. The differences in time within individuals are sometimes greater than those between individuals. For example, in the first axis, the distance between Koala id 5 at T0 and Koala id 3 at time 5 days is 0.01, while the distance between Koala id 3 at T0 and Koala id 5 at T0 was 0.14. Thus, except for Koala id 1, time substantially affected the presence-absence of fungal composition measured as a function of Jaccard distance (Supplementary Figure 4).

**Supplementary Figures and Tables**
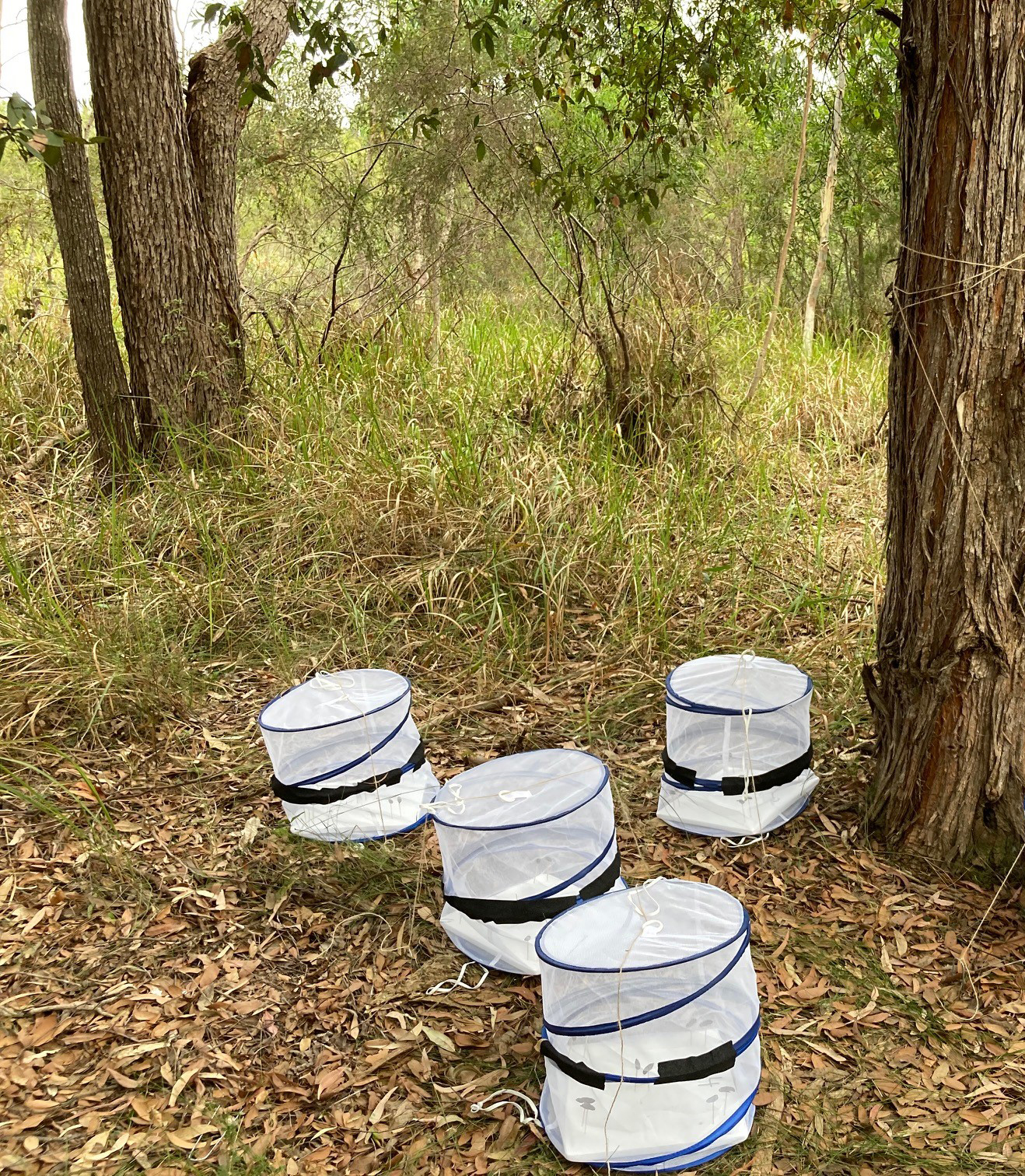


**Supplementary Figure 1.** **Picture of** **Scat aging setup**: five scat pellets for each koala were then mounted on toothpicks, suspended on Styrofoam trays, and placed into meshed enclosures and aged under natural conditions in a remnant patch of forest on campus (University of sunshine Coast).

**
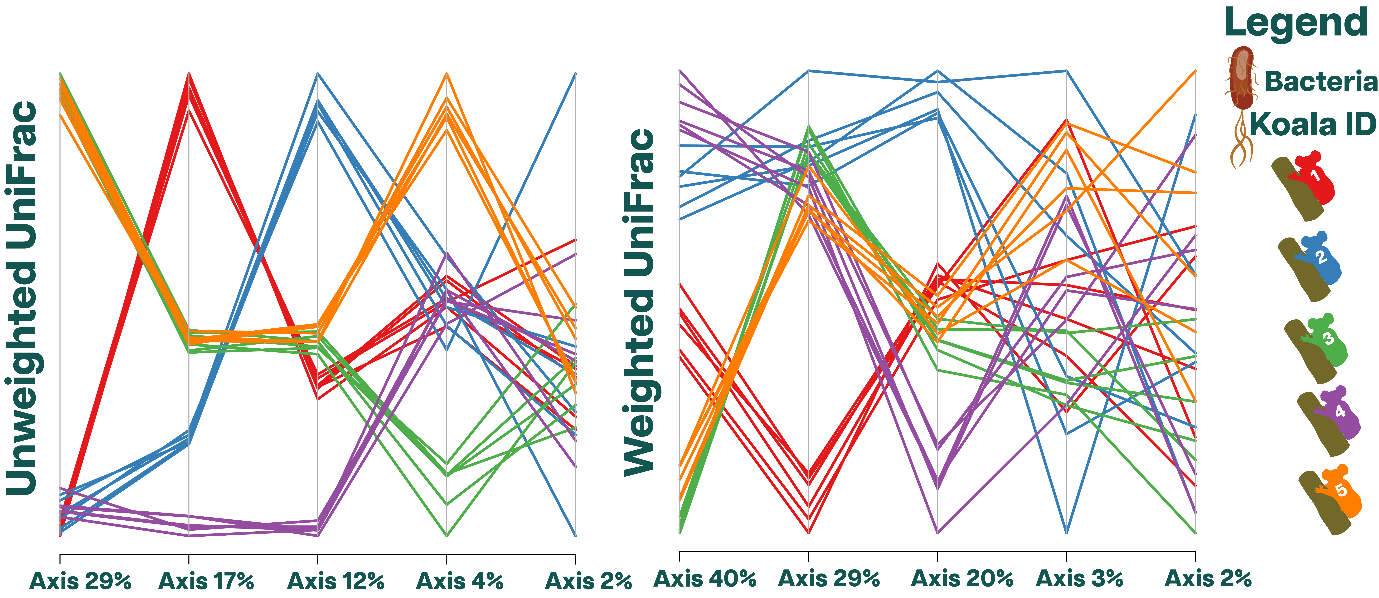
**

B

A

**Supplementary Figure 2. Phylogenetic beta diversity plots for bacteria.** The scat samples taken from five koalas are represented by six points in time from 0 to 10 days. Therefore, each colour denotes a koala id (color-coded in the plot) with six lines representing those time points. The first five principal axes were plotted, and the total amount of variance they account for was printed on the x-axis. Plot (A) Unweighted UniFrac and plot (B) Weighted UniFrac.

**
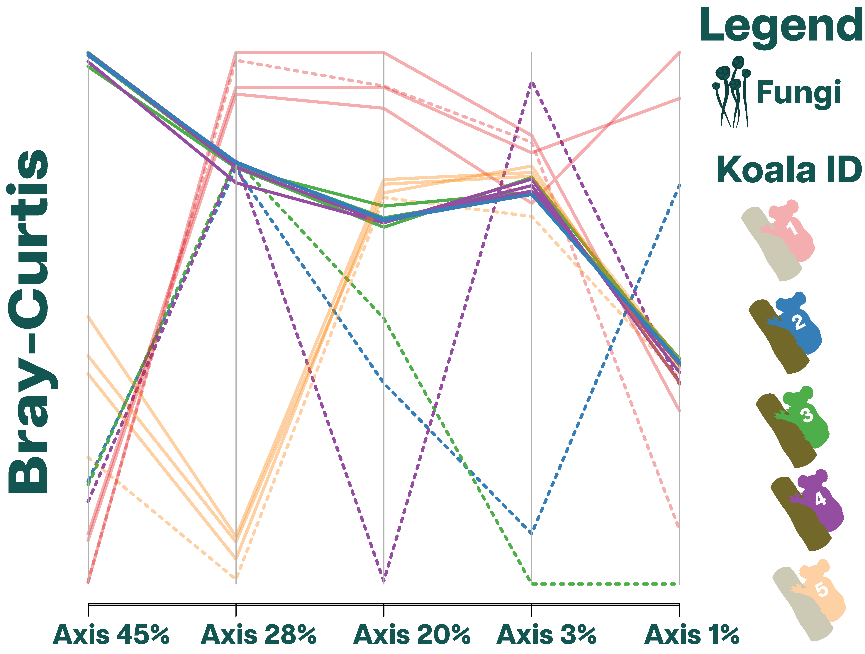
**

**Supplementary Figure 3. Effect of time after 72 hrs of scat aging in fungi.** Bray Curtis dissimilarity principal coordinates analysis represented by a parallel coordinates plot. The scat samples were taken from five koalas; each denotes a koala ID (color-coded in the plot). The first five principal axes were plotted, and the total amount of variance they account for was printed on the x-axis. The dashed lines represent the T0 samples, while the solid lines represent samples at 72hr, 5 days, and 10 days of scat aging. The koalas most affected by time from an abundance perspective are represented by the opaque lines, while the two koalas less affected by time are represented by the lower opacity lines.

**
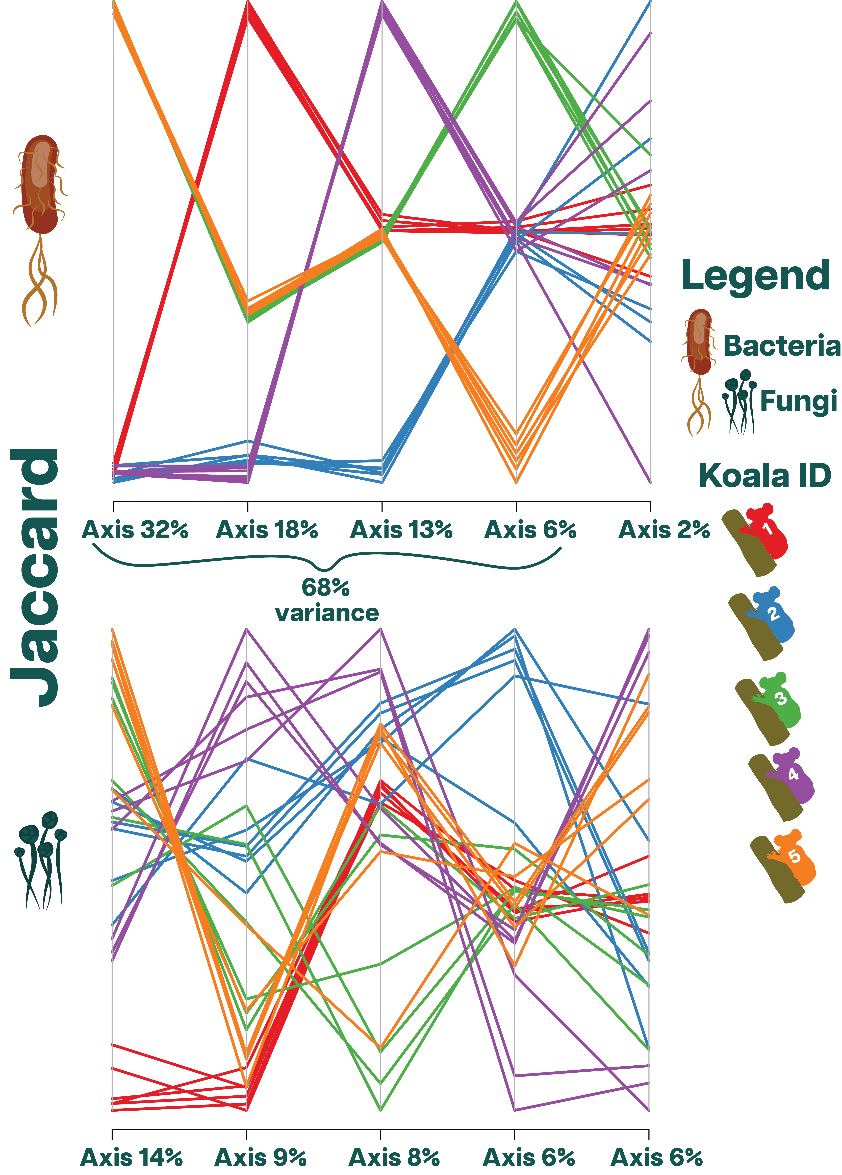
**

B

A

**Supplementary Figure 4.** **Comparison of beta diversity between koalas.** Jaccard dissimilarity principal coordinates analysis represented by a parallel coordinates plot. The scat samples taken from five koalas are represented by six points in time from 0 to 10 days. Therefore, each color denotes a koala id (color-coded in the plot) with six lines representing those time points. The first five principal axes were plotted, and the total amount of variance they account for was printed on the x-axis. Plot (**A**) accounts for bacteria (V3 and V4), and plot (**B**) accounts for Fungi (ITS1 and ITS2).


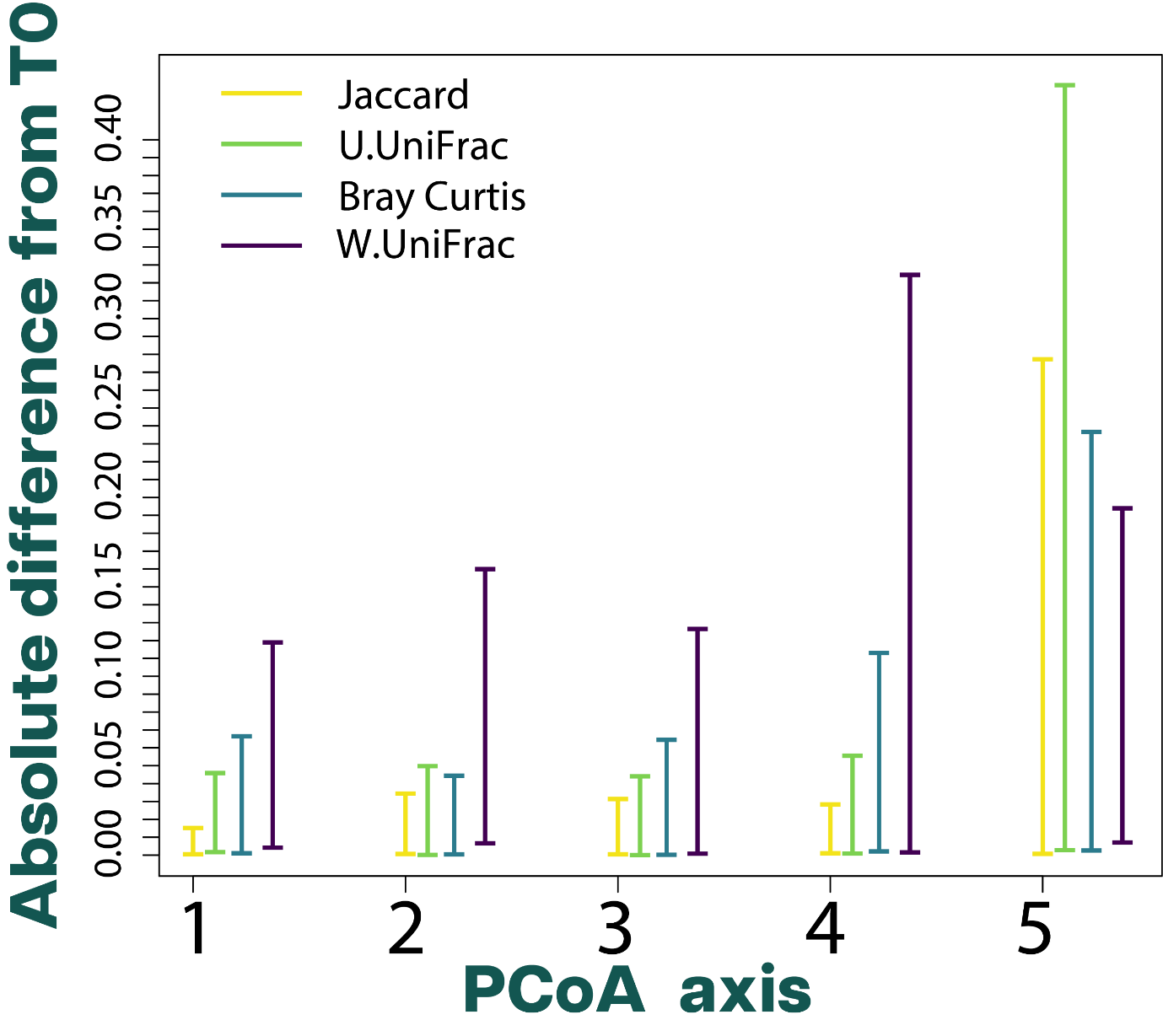


**Supplementary Figure 5. The effect of scat aging on bacterial beta diversity metrics.** The graph represents the range of absolute difference between T0 and the remaining timepoints for the first five PCoA axes for four different beta diversity metrics.

**Supplementary Table 1 Bacteria Quality control metrics.** Number of reads that made it through each step of dada2 pipeline

| sample-id | input | filtered | denoised | non-chimeric |
| --- | --- | --- | --- | --- |
| 1A_T0_B-16S_V3-V4 | 178924 | 154112 | 152161 | 102123 |
| 1A_T10_B-16S_V3-V4 | 178392 | 152396 | 151007 | 116359 |
| 1A_T24_B-16S_V3-V4 | 127988 | 110083 | 108943 | 77468 |
| 1A_T48_B-16S_V3-V4 | 196277 | 171296 | 169945 | 124051 |
| 1A_T5_B-16S_V3-V4 | 183131 | 160000 | 158329 | 112348 |
| 1A_T72_B-16S_V3-V4 | 142867 | 122860 | 121611 | 91765 |
| 2A_T0_B-16S_V3-V4 | 160376 | 137091 | 135202 | 104012 |
| 2A_T10_B-16S_V3-V4 | 126850 | 106489 | 105083 | 78377 |
| 2A_T24_B-16S_V3-V4 | 122045 | 105315 | 103854 | 77113 |
| 2A_T48_B-16S_V3-V4 | 134006 | 115684 | 114161 | 86147 |
| 2A_T5_B-16S_V3-V4 | 199514 | 170759 | 168904 | 126926 |
| 2A_T72_B-16S_V3-V4 | 153633 | 131606 | 130255 | 102337 |
| 3A_T0_B-16S_V3-V4 | 164493 | 141198 | 139215 | 96118 |
| 3A_T10_B-16S_V3-V4 | 143941 | 121208 | 120064 | 91193 |
| 3A_T24_B-16S_V3-V4 | 153980 | 131742 | 130076 | 93520 |
| 3A_T48_B-16S_V3-V4 | 160261 | 136978 | 135342 | 95414 |
| 3A_T5_B-16S_V3-V4 | 139199 | 118770 | 117262 | 83959 |
| 3A_T72_B-16S_V3-V4 | 177899 | 152793 | 150903 | 111611 |
| 4A_T0_B-16S_V3-V4 | 163444 | 140788 | 139304 | 99856 |
| 4A_T10_B-16S_V3-V4 | 158387 | 136870 | 135797 | 102055 |
| 4A_T24_B-16S_V3-V4 | 147820 | 127112 | 125480 | 85285 |
| 4A_T48_B-16S_V3-V4 | 129797 | 112215 | 111383 | 86246 |
| 4A_T5_B-16S_V3-V4 | 216231 | 186519 | 185161 | 145189 |
| 4A_T72_B-16S_V3-V4 | 239633 | 202969 | 201123 | 142798 |
| 5A_T0_B-16S_V3-V4 | 174336 | 148691 | 147121 | 106660 |
| 5A_T10_B-16S_V3-V4 | 208094 | 179105 | 177471 | 137869 |
| 5A_T24_B-16S_V3-V4 | 172655 | 149503 | 147688 | 108123 |
| 5A_T48_B-16S_V3-V4 | 134331 | 112113 | 110878 | 84404 |
| 5A_T5_B-16S_V3-V4 | 157995 | 137007 | 135590 | 102218 |
| 5A_T72_B-16S_V3-V4 | 199820 | 172072 | 169929 | 124777 |

**Supplementary Table 2 Fungi Quality control metrics**. Number of reads that made it through each step of dada2 pipeline

| sample-id | input | filtered | denoised | non-chimeric |
| --- | --- | --- | --- | --- |
| 1A_T0_F-ITS | 77052 | 67145 | 66594 | 25863 |
| 1A_T10_F-ITS | 40370 | 35353 | 35008 | 22005 |
| 1A_T24_F-ITS | 73424 | 64616 | 64166 | 25929 |
| 1A_T48_F-ITS | 83653 | 72999 | 72502 | 28523 |
| 1A_T5_F-ITS | 87831 | 76483 | 75930 | 35053 |
| 1A_T72_F-ITS | 17186 | 13704 | 13503 | 8349 |
| 2A_T0_F-ITS | 13211 | 10134 | 9971 | 6734 |
| 2A_T10_F-ITS | 133670 | 118039 | 117944 | 117785 |
| 2A_T24_F-ITS | 116721 | 100959 | 100876 | 100657 |
| 2A_T48_F-ITS | 101930 | 88685 | 88616 | 88585 |
| 2A_T5_F-ITS | 164822 | 144795 | 144583 | 142832 |
| 2A_T72_F-ITS | 135815 | 120267 | 120182 | 120011 |
| 3A_T0_F-ITS | 83489 | 71598 | 70787 | 31230 |
| 3A_T10_F-ITS | 144495 | 127742 | 127648 | 127325 |
| 3A_T24_F-ITS | 142159 | 124235 | 124024 | 119510 |
| 3A_T48_F-ITS | 128274 | 110171 | 109739 | 100799 |
| 3A_T5_F-ITS | 140232 | 120402 | 120109 | 116743 |
| 3A_T72_F-ITS | 172205 | 149864 | 149751 | 149301 |
| 4A_T0_F-ITS | 76090 | 66294 | 65954 | 26327 |
| 4A_T10_F-ITS | 142926 | 123271 | 123141 | 121434 |
| 4A_T24_F-ITS | 73002 | 63858 | 63482 | 33286 |
| 4A_T48_F-ITS | 60488 | 50620 | 50275 | 21438 |
| 4A_T5_F-ITS | 145878 | 126684 | 126532 | 123668 |
| 4A_T72_F-ITS | 193888 | 166804 | 166646 | 166246 |
| 5A_T0_F-ITS | 127388 | 112303 | 112013 | 108890 |
| 5A_T10_F-ITS | 159077 | 138588 | 138418 | 136523 |
| 5A_T24_F-ITS | 162149 | 144955 | 144864 | 143008 |
| 5A_T48_F-ITS | 142483 | 128581 | 128490 | 128486 |
| 5A_T5_F-ITS | 122447 | 110072 | 109971 | 109371 |
| 5A_T72_F-ITS | 140319 | 123585 | 123499 | 123116 |

**Supplementary Table 3 Respiration profile of 27 most abundant taxonomic families for bacteria.**

| Taxonomic Family | Respiration |
| --- | --- |
| Lachnospiraceae | Obligate Anaerobe |
| Ruminococcaceae | Obligate Anaerobe |
| Oscillospiraceae | Obligate Anaerobe |
| Bacteroidaceae | Obligate Anaerobe |
| Synergistaceae | Anaerobe |
| Tannerellaceae | Obligate Anaerobe |
| Enterobacteriaceae | Facultative Anaerobe |
| Acidaminococcaceae | Anaerobe |
| Rikenellaceae | Anaerobe |
| Monoglobaceae | Obligate Anaerobe |
| Pasteurellaceae | Facultative Anaerobe |
| Desulfovibrionaceae | Anaerobe |
| Eggerthellaceae | Anaerobe |
| Selenomonadaceae | Anaerobe |
| Fusobacteriaceae | Anaerobe |
| Sutterellaceae | Microaerophile |
| Erysipelatoclostridiaceae | Facultative Anaerobe |
| Coriobacteriaceae | Anaerobe |
| Syntrophomonadaceae | Anaerobe |
| Christensenellaceae | Anaerobe |
| Marinifilaceae | Anaerobe |
| Paludibacteraceae | Anaerobe |
| Enterococcaceae | Facultative Anaerobe |
| Anaerovoracaceae | Anaerobe |
| Deferribacteraceae | Anaerobe |
| Dysgonomonadaceae | Anaerobe |
| Prevotellaceae | Anaerobe |
